# Desmosine in Aortic Disease: Biology, Measurement, and Clinical Applications in Aortic Pathologies

**DOI:** 10.3390/jcm15072540

**Published:** 2026-03-26

**Authors:** Alexander Gombert, Saurav Ranjan Mohapatra, Jelle M. Frankort, Christian Uhl, Panagiotis Doukas

**Affiliations:** Department of Vascular Surgery, University Hospital RWTH Aachen, 52074 Aachen, Germany; samohapatra@ukaachen.de (S.R.M.); jefrankort@ukaachen.de (J.M.F.); cuhl@ukaachen.de (C.U.);

**Keywords:** desmosine, elastin, aneurysm, risk prediction, biomarkers

## Abstract

Thoracoabdominal aortic aneurysms (TAAAs) are uncommon and usually silent until rupture, causing a substantial burden to the health care system. Aneurysm growth and rupture prediction is mainly based on aneurysm diameter measurement by imaging modalities, meaning that the biology of aneurysm growth is not part of a potentially more adequate surveillance of aortic aneurysm patients. Alternatives or complementary options for aortic aneurysm surveillance are an ongoing, non-addressed open issue of vascular medicine. The application of different biomarkers has been discussed, yet so far, an adequate candidate for aortic aneurysm surveillance, if it comes to the thoracic or thoracoabdominal aorta, preferably without radiation exposure, has not been named. Elastin breakdown, as a component of aortic wall degeneration primarily driven by matrix metalloproteinases (MMPs), is a core element of aneurysm development. Desmosine is an elastin-specific cross-link increasingly studied as a circulating or urinary biomarker of compromised aortic wall integrity and disease activity. Accordingly, this review investigated whether plasma desmosine (pDES), a highly specific marker of elastin degradation, could be used as a non-invasive biomarker for detecting aortic aneurysms and assessing their risk profile. The existing literature of desmosine in fields of aortic pathologies in the acute and chronic setting will be assessed based on the current literature; furthermore, future perspectives of desmosine as a biomarker of aortic pathologies, such as aortic aneurysm dynamics, will be discussed.

## 1. Introduction

### 1.1. General Biology of Desmosine and Elastin

Elastin is the key load-bearing protein that provides resilience and recoil to large arteries, particularly the aorta. Its mature fibers are stabilized by unique tetrafunctional cross-linking amino acids, desmosine and isodesmosine, formed during post-translational modification of tropoelastin [1].

The fragmentation of elastin is closely related to medial calcification: Warfarin-induced elastocalcinosis shows MMP-9 and TGF-β activation as well as calcification later on, indicating matrix degradation precedes overt mineral deposition [2].

In a uremic mouse chronic kidney disease model, elastin disorganization and breaks are present by week 4 before arterial medial calcification (AMC) appears, and then calcification localizes to regions of severe elastin breaks [3].

The aorta of Marfan patients shows microcalcification co-localized with elastic lamina fragmentation, and elastin peptides directly induce calcification signaling (in smooth muscle cells, SMCs) via the elastin receptor/ERK1/2 pathway [4].

Elastolytic proteases such as neutrophil elastase and MMP-12 liberate desmosine and isodesmosine from insoluble elastin as free amino acids, especially when elastin has been oxidatively modified. This mechanistic link underpins the use of circulating or urinary desmosine as a specific marker of mature elastin degradation in vascular disease. Tissue-level work in aortic aneurysms and dissections consistently shows pronounced loss or modification of elastin cross-links. Abdominal aortic aneurysm (AAA) walls display about a 90% reduction in desmosine/isodesmosine compared with non-aneurysmal aorta, alongside increased collagen cross-links, indicating profound elastin depletion and matrix remodeling [5,6]. In dissecting aneurysm of the thoracic aorta, all measured elastin cross-links (desmosine, isodesmosine, neodesmosine, oxodesmosine) are reduced, with particularly marked loss of oxidized derivatives, suggesting oxidative damage and cross-link breakdown in disease pathogenesis [7].

### 1.2. Analytical Methods and Measurement Considerations

Quantification of desmosine has evolved from immunoassays and HPLC to highly specific stable-isotope dilution LC–MS/MS assays, now the standard for plasma and urine measurements [8,9,10]. These techniques allow separation of desmosine and isodesmosine, low ng/mL sensitivity, and robust quantification suitable for multicenter clinical studies.

Specimen choice is important. Plasma desmosine (pDES) reflects systemic elastin degradation from arteries, lung, and other elastin-rich tissues, whereas urinary desmosine (uDES), often normalized to creatinine, integrates filtered and excreted cross-links over time and may be more sensitive to chronic aortic wall injury in some conditions [11]. Renal function, age, smoking, and coexistent pulmonary disease can influence circulating levels and must be considered as potential confounders [1,12].

## 2. Current Evidence

### 2.1. Desmosine and Lung Disease

Desmosine and isodesmosine (DES) are amino acids found exclusively in cross-linked elastin fibers, so circulating DES levels serve as an indicator of elastin degradation [13]. The COPD Biomarker Qualification Consortium has highlighted DES as a biomarker with substantial potential for COPD [14].

In a clinically homogeneous cohort of COPD patients with alpha-1 antitrypsin (AAT) deficiency, higher plasma DES concentrations were associated with progression of emphysema. In contrast, in a broader COPD cohort encompassing multiple endotypes and phenotypes, plasma DES did not correlate with emphysema severity.

In this more heterogeneous COPD population, plasma DES levels were instead linked to arterial stiffness as well as aortic-femoral pulse wave velocity and cardiovascular comorbidities [12], suggesting that, in most AAT-sufficient COPD patients, the vasculature is the predominant source of elastin degradation products entering the circulation. Notably, plasma DES was also associated with coronary artery calcium (CAC) score, underscoring a close connection between elastinolysis and vascular calcification [12].

### 2.2. Desmosine and Cerebral Artery Aneurysm

In their paper, Csécsei et al. assessed a single-center prospective study cohort of 135 adults with Aneurysmal Subarachnoid Hemorrhage (aSAH) and 25 matched healthy controls, excluding conditions known to raise desmosine (e.g., COPD, abdominal aortic aneurysm, malignancy, major organ disease) [15]. Serum desmosine was measured and correlated with aneurysm size, neck width, aspect ratio, and size ratio, which were quantified from 3D digital subtraction angiography, and clinical severity and outcome were scored with standard scales (WFNS, modified Fisher, 3-month mRS). They observed higher desmosine in aSAH vs. controls and an association with aneurysm size, which correlated positively with desmosine. All this leads to a diagnostic performance with an AUC of 0.804 (95% CI 0.723–0.884); an optimal cut-off of 0.533 ng/mL yielded a sensitivity 81.7% and specificity 60.3%. Yet, no correlation with the WFNS score and modified Fisher score could be observed.

### 2.3. Desmosine in Abdominal Aortic Aneurysm

**Figure 1:**
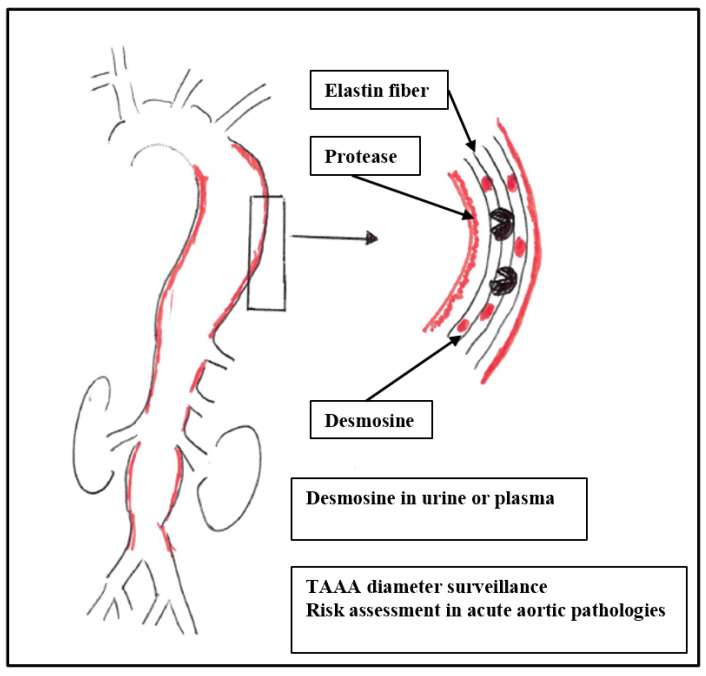
Application of Desmosine as biomarker for thoracoabdominal aortic aneurysm growth.

AAA development is closely linked to the breakdown of elastin within the tunica media. Histochemical and biochemical studies show severe loss of elastin content and cross-links, with up to ~90% reduction in desmosine/isodesmosine in aneurysmal tissue compared with controls [5,6] (Figure 1). Elastin mRNA remains detectable, suggesting ongoing but insufficient repair amid overwhelming degradation [16]. Large cohort studies demonstrate that circulating pDES is elevated in AAA and relates to disease severity and prognosis; in their study, Mordi et al. assessed 507 patients with AAA versus 162 controls. Here, pDES was higher in AAA (0.46 ± 0.22 vs. 0.33 ± 0.16 ng/mL) and had the strongest correlation with aneurysm diameter among tested biomarkers (r ≈ 0.39) [8]. A follow-up study by the same research group showed that during the follow-up of n = 239 patients, a higher baseline pDES independently predicted AAA events (rupture, repair, AAA-related death) after adjustment for diameter (HR ≈ 2.0 per SD increase) and modestly improved risk reclassification beyond size alone [8]. Another prospective study correlated pDES with ultrasound diameter (r = 0.27) and independently predicted emergency AAA events and broader cardiovascular outcomes (HR ≈ 5 for the highest vs. the lowest tertiles), outperforming MMP-9 for event prediction.

These data support pDES as a marker of aneurysm activity and short- to mid-term risk, complementing but not replacing diameter-based surveillance.

### 2.4. Desmosine in Thoracoabdominal Aortic Aneurysm

In their paper, Doukas et al. evaluate plasma desmosine as a blood marker of elastin breakdown in thoracoabdominal aortic aneurysm (TAAA) and link it to molecular changes in the aortic wall. 30 patients scheduled for open TAAA repair, and 30 age- and sex-matched healthy controls were prospectively enrolled, plasma desmosine (pDES) was measured using a validated LC-MS/MS assay, while full-thickness aortic wall samples from 12 patients were analyzed histologically for elastin content and by Western blot for matrix metalloproteinases (MMPs) and tissue inhibitor TIMP-1. TAAA patients had substantially higher pDES than controls (0.40 ± 0.31 vs. 0.22 ± 0.15 ng/mL, *p* < 0.001). In aneurysm tissue, pDES correlated positively with aortic wall MMP-2 and TIMP-1 expression and with the proportion of medial elastic fibers, indicating that circulating pDES reflects intramural proteolytic activity and elastin content. Diagnostic performance analysis showed an AUC of 0.82 for pDES in distinguishing TAAA from controls; a cut-off of 0.27 ng/mL achieved 78.6% sensitivity and 76.7% specificity [17]. Notably, pDES did not correlate with maximum aortic diameter, suggesting it captures the biological activity of the aneurysm rather than size alone [17]. Overall, the paper concludes that elevated pDES mirrors MMP-mediated elastin degradation in TAAA and could serve as a non-invasive biomarker for screening and risk assessment in patients with thoracoabdominal aortic aneurysms, warranting validation in larger cohorts.

### 2.5. Desmosine in Thoracic Aortic and Heritable Aortopathies

Iskendar et al. assessed how elastin breakdown changes with age in people with Marfan syndrome (MFS) compared with healthy controls, and how this relates to aortic root size, using plasma desmosine (pDES) as a specific biomarker of mature elastin degradation [18]. In their post hoc analysis of the Aortic Irbesartan Marfan Study (AIMS), including 113 MFS patients (ages 6–40) and 109 healthy controls, grouped into three developmental stages: 6–15, >15–25, and >25–40 years 1.2. pDES was quantified by LC–MS/MS, and aortic root diameters were measured longitudinally (up to 5 years in MFS) by echocardiography or MRI [19]. In their study, pDES was higher in children and fell to lower, stable levels in adults in both groups, while this age effect was exaggerated in MFS, with significantly higher pDES than controls in the 6–15 and 15–25 age ranges, but not after 25 years. Accordingly, the authors stated an association between higher pDES and larger aortic diameter, especially before 15 years of age, which diminished in later age groups. Over up to 5 years of follow-up, baseline pDES showed a suggestive but not statistically significant association with aortic dilatation rate (adjusted effect 0.18 mm/year per 0.5 ng/mL pDES, *p* = 0.10). In adults > 25 years with established aortic root dilation, pDES levels were not elevated compared with controls, suggesting that further aortic enlargement in adulthood may be driven more by altered aortic mechanics and vascular aging than by active elastin degradation. In conclusion, developmental age was judged as strongly relevant for elastin turnover; in MFS, elastin degradation is excessively high in childhood and adolescence, coinciding with rapid aortic growth and early aortopathy.

### 2.6. Bicuspid Aortic Valve–Associated Aortopathy

Patients with bicuspid aortic valve (BAV) frequently develop ascending aortic dilatation characterized by matrix remodeling and elastin degradation.

In a cohort of 20 BAV patients vs. age-matched controls, both plasma Desmosine (pDES) and urinary Desmosine (uDES) were significantly higher in BAV (e.g., uDES 15.9 ± 4.6 vs. 7.2 ± 2.8 ng/mg creatinine [11]. In this study, Urinary desmosine showed strong correlations with aortic root diameter (r ≈ 0.6–0.65) and z-scores, whereas pDES correlations were weaker and not statistically significant in this small sample. These findings suggest uDES may serve as a non-invasive marker of ascending aortic wall elastin injury and dilatation in BAV, although larger longitudinal studies are required.

### 2.7. Acute Aortic Syndromes

In acute aortic syndromes (AAS; dissection, intramural hematoma, penetrating ulcer), rapid elastin disruption occurs at the time of wall failure. Syed et al. measured pDES in 53 patients with radiologically confirmed AAS vs. 106 controls; here, pDES was almost doubled (0.58 ± 0.26 vs. 0.27 ± 0.07 ng/mL) [20]. pDES was highest at presentation and declined with time from the index event (negative correlation with time since AAS), consistent with an acute surge from intense elastin breakdown [20]. Among AAS patients, pDES was the only measured variable significantly associated with progressive aortic enlargement over follow-up, supporting a potential role in early diagnosis and risk stratification. Serum biomarker reviews of aortic dissection highlight soluble elastin fragments among promising tools for rapid differentiation from other acute chest pain syndromes, although most focus on broader elastin peptides rather than desmosine specifically [21]. Desmosine’s high specificity for mature elastin breakdown makes it an attractive candidate marker within this group.

### 2.8. Desmosine and Systemic Atherosclerotic Burden

Beyond focal aneurysm disease, pDES tracks generalized vascular elastin degradation.

In patients from the SUMMIT study, pDES was higher in those with established cardiovascular disease and showed a strong independent association with whole-body atheroma burden measured by standardized atheroma score on whole-body MRA (adjusted β ≈ 19, *p* = 0.004) [9].

In COPD cohorts, pDES is elevated in those with cardiovascular comorbidity, correlates with coronary calcium, arterial stiffness, and systemic inflammation, and independently predicts all-cause mortality, strengthening its link to vascular risk rather than lung destruction alone [12].

After acute myocardial infarction, higher pDES is associated with adverse outcomes (death or recurrent MI) at follow-up and can improve risk classification when added to established scores.

These data position desmosine as a global indicator of arterial elastin damage and cardiovascular risk, which is pathobiologically relevant to both aneurysmal and occlusive aortic disease.

### 2.9. Mechanistic Insights from Experimental Work

In vitro studies provide mechanistic support for clinical associations as described before.

Elastolytic proteases implicated in arterial disease (MMP-12, neutrophil elastase) efficiently liberate free desmosine and isodesmosine from insoluble elastin, especially when elastin has been pre-oxidized by reactive oxygen species.

This observation implies that inflammatory and oxidative processes that drive aortic aneurysm formation and growth, as well as aortic dissection, will simultaneously generate measurable increases in circulating or urinary desmosine, linking biochemical release to pathologic remodeling in the aortic wall.

Tissue-level analyses in AAA and dissecting aneurysm show profound reductions and oxidative modifications of elastin cross-links, consistent with chronic and acute destructive processes that would be expected to raise circulating desmosine levels [5,6].

Desmosine mechanistically reflects covalent crosslinks lost during elastin fiber failure. This explains its tight links with structural lung damage, vascular disease, and mortality, and supports its role as a mechanistic biomarker to bridge molecular elastin turnover with clinically meaningful outcomes. When elastin is damaged, these crosslinks are released and can be measured, making them attractive for mechanistic studies of emphysema and COPD, as described in the mentioned literature [12,17]. Yet, currently, more specific research projects assessing the release of pDES or uDES in fields of vascular or pulmonary research are pending.

## 3. Discussion

### 3.1. Clinical Applications and Limitations in Aortic Pathologies

Thoracoabdominal aortic aneurysms (TAAAs) are a serious health care issue, causing a substantial burden to the health care system [22]. Aneurysm growth and rupture prediction is mainly based on aneurysm diameter measurement by imaging modalities, meaning that the biology of aneurysm growth is not part of a potentially more adequate surveillance of aortic aneurysm patients.

As addressed in the current DTAA guideline of the ESVS, the detection of acute aortic pathologies and aneurysm growth prediction are open issues that should be addressed in future research projects; so far, no real candidates as suitable biomarkers for this surveillance have been identified in the field of clinical application [23].

Regardless, as clearly underlined in the current guideline of the ESVS, more sophisticated and less invasive tools are urgently required to improve surveillance during follow-up and as decision-making support in the acute setting of aortic pathologies. Desmosine shows a lot of potential to be applied as a biomarker for the acute and chronic aortic setting, and coupled with an established, predefined cut-off could meaningfully support clinical decision making. With a direct link to elastolytic degradation of the aortic wall, pDES measurements could stratify aneurysms according to proteolytic activity and discern the patients at risk of adverse aortic events. In fields of acute aortic syndrome, such as acute type B dissection or aortic intramural hematoma, pDES levels could help identify the cases that warrant early invasive therapy to improve prognosis. In chronic cases, they could complement current imaging-based surveillance protocols, such as computed tomography and MRI scans. Such an approach could contribute to reducing cumulative radiation and contrast exposure, thereby offering potential economic advantages, while also providing an additional consideration in the algorithm for treatment indication in the increasingly elderly and frail TAAA population.

In general, across aortic disease spectra, current evidence supports several potential roles, such as AAA surveillance and risk stratification: pDES adds prognostic information beyond diameter for predicting rupture/repair events and may refine thresholds for imaging frequency or timing of intervention [8]. In fields of BAV and heritable aortopathies, uDES (and to a lesser extent pDES) may reflect ascending aortic dilatation and elastin degradation and could serve as a non-invasive marker to complement imaging during long-term follow-up

As mentioned, elevated pDES levels in the case of Acute aortic syndromes could be measured at presentation. A subsequent decline suggests potential diagnostic and prognostic utility in the acute setting and for monitoring post-event aortic remodeling [20].

Regarding global vascular risk, it could be observed that in patients with systemic atherosclerosis, COPD, CAD, or after AMI, pDES may help identify individuals with high vascular elastin turnover and elevated risk of adverse cardiovascular outcomes [9,12,18].

### 3.2. Limitations and Research Gaps

Several important caveats limit immediate routine clinical use until today:

A relevant lack regarding the specificity of the source has to be mentioned, as pDES reflects total body elastin degradation (lung, skin, large arteries). So, comorbid pulmonary disease and aging can confound interpretation in aortic disease [1,12]. Standardization is an issue, although LC–MS/MS methods are well established. Yet there is no universally accepted cut-off or reference range across ages, sexes, and comorbidities; pre-analytical conditions and normalization (particularly for urine) need harmonization [8,10]. The available studies are single-center cohorts and include low numbers of patients with limited data on intra-individual variability, response to therapy, and incremental value in decision-making beyond imaging [11,17,20]. Finally, while desmosine robustly reflects elastin breakdown, it does not identify upstream drivers (genetic vs. inflammatory vs. hemodynamic) or distinguish between stable remodeling and imminent rupture. Furthermore, Smoking (including passive and former exposure) alters desmosine levels and is correlated with key outcomes, so it should be treated and adjusted for as a confounding variable in desmosine biomarker studies [24].

### 3.3. Future Directions

Desmosine release from aortic tissue could be assessed by mechanistic investigations linking local protease activity, oxidative stress, and regional wall biomechanics in human aorta, supported by experimental models [1,6,7]. Large, prospective, multicenter cohorts integrating pDES/uDES with advanced imaging (4D flow MRI, PET, radiomics) to build multimodal risk scores for AAA, BAV aortopathy, and post-dissection remodeling [8] could strengthen the assumed meaning of desmosine as a predictor of aortic aneurysm growth and acute aortic pathologies by study design and appropriate power calculation. Studies in genetically mediated aortopathies (e.g., Marfan, Loeys–Dietz) could help to clarify whether desmosine levels track genotype, growth rates, and outcomes and to define disease-specific thresholds. A user-friendly bedside-testing kit would enable emergency and elective assessment of desmosine plasma or urine levels, which would be most favorable regarding clinical application in daily practice and prospective studies in different medical fields. Yet, so far, the measurement is sophisticated and is not part of regular laboratory tests, which is an obvious obstacle for broader utilization and clinical evaluation. The lack of a clinically available, easy-to-use and swift measurement system is a relevant limitation of the ability of pDES or uDES as a biomarker. Furthermore, the already mentioned confounders, regarding aortic pathologies, such as lung disease and, to an unknown level, smoking, require more research to evaluate the applicability of desmosine for patients in a real-life scenario.

Interventional trials assessing whether medical therapies that slow aneurysm growth or reduce inflammation also lower desmosine and whether biomarker changes predict treatment response.

## 4. Conclusions

Desmosine is a structurally and mechanistically specific marker of mature elastin degradation with growing evidence across the spectrum of aortic disease. Elevated circulating and urinary desmosine may be associated with the presence and severity of different acute and chronic aortic pathologies and may predict aneurysm-related and broader cardiovascular events independently of traditional risk factors and aortic size. Standardized measurement and larger prospective studies are needed before desmosine can be incorporated into routine clinical algorithms, but current data indicate substantial promise as a complementary biomarker for diagnosis, surveillance, and risk stratification in aortic pathologies.

## Data Availability

The data used for this review are available upon request.
